# Polypyrrole-Wrapped Carbon Nanotube Composite Films Coated on Diazonium-Modified Flexible ITO Sheets for the Electroanalysis of Heavy Metal Ions

**DOI:** 10.3390/s20030580

**Published:** 2020-01-21

**Authors:** Momath Lo, Mahamadou Seydou, Asma Bensghaïer, Rémy Pires, Diariatou Gningue-Sall, Jean-Jacques Aaron, Zineb Mekhalif, Joseph Delhalle, Mohamed M. Chehimi

**Affiliations:** 1 Faculté des Sciences, Université Cheikh Anta Diop, BP 5005 Dakar-Fann, Senegal; diariatougningue.sall@ucad.edu.sn; 2 Laboratoire Géomatériaux et Environnement, Université Paris-Est, 5 Bd. Descartes, CEDEX 2, 77454 Marne-la-Vallée, France; 3 University Paris Est Creteil, CNRS, ICMPE, UMR7182, F-94320 Thiais, France; bensghaier@icmpe.cnrs.fr (A.B.); pires@icmpe.cnrs.fr (R.P.); 4 Université de Paris, ITODYS, CNRS, UMR 7086, 15 rue J-A de Baïf, F-75013 Paris, France; mahamadou.seydou@univ-paris-diderot.fr; 5 Laboratory of Chemistry and Electrochemistry of Surfaces (CES), University of Namur, rue de Bruxelles, 61, B-5000 Namur, Belgium; zineb.mekhalif@unamur.be (Z.M.); joseph.delhalle@unamur.be (J.D.)

**Keywords:** polypyrrole, diazonium, multiwalled carbon nanotubes, chelator, heavy metal ions, electrochemical sensors

## Abstract

Highly sensitive multicomponent materials designed for the recognition of hazardous compounds request control over interfacial chemistry. The latter is a key parameter in the construction of the sensing (macro) molecular architectures. In this work, multi-walled carbon nanotubes (CNTs) were deposited on diazonium-modified, flexible indium tin oxide (ITO) electrodes prior to the electropolymerization of pyrrole. This three-step process, including diazonium electroreduction, the deposition of CNTs and electropolymerization, provided adhesively-bonded, polypyrrole-wrapped CNT composite coatings on aminophenyl-modified flexible ITO sheets. The aminophenyl (AP) groups were attached to ITO by electroreduction of the in-situ generated aminobenzenediazonium compound in aqueous, acidic medium. For the first time, polypyrrole (PPy) was electrodeposited in the presence of both benzenesulfonic acid (dopant) and ethylene glycol-bis(2-aminoethylether)-tetraacetic acid (EGTA), which acts as a chelator. The flexible electrodes were characterized by XPS, Raman and scanning electron microscopy (SEM), which provided strong supporting evidence for the wrapping of CNTs by the electrodeposited PPy. Indeed, the CNT average diameter increased from 18 ± 2.6 nm to 27 ± 4.8, 35.6 ± 5.9 and 175 ± 20.1 after 1, 5 and 10 of electropolymerization of pyrrole, respectively. The PPy/CNT/NH_2_-ITO films generated by this strategy exhibit significantly improved stability and higher conductivity compared to a similar PPy coating without any embedded CNTs, as assessed by from electrochemical impedance spectroscopy measurements. The potentiometric response was linear in the 10^−8^–3 × 10^−7^ mol L^−1^ Pb(II) concentration range, and the detection limit was 2.9 × 10^−9^ mol L^−1^ at S/N = 3. The EGTA was found to drastically improve selectivity for Pb(II) over Cu(II). To account for this improvement, the density functional theory (DFT) was employed to calculate the EGTA–metal ion interaction energy, which was found to be −374.6 and −116.4 kJ/mol for Pb(II) and Cu(II), respectively, considering solvation effects. This work demonstrates the power of a subtle combination of diazonium coupling agent, CNTs, chelators and conductive polymers to design high-performance electrochemical sensors for environmental applications.

## 1. Introduction

Extensive research on carbon nanotubes (CNTs) in the fields of applied physics, chemistry, and materials science and engineering has progressed at a remarkable pace owing to CNTs’ outstanding mechanical properties, good electronic conductivity, 1D structure, nanometer size diameter and high-accessible surface area [1]. In order to design ultra-sensitive sensors, CNTs have been used either as such, or after modification by a variety of ways [2,3,4,5].

Conductive polymers (CPs), including polypyrrole (PPy), polyaniline (PANI) and polythiophene (PT), constitute a class of materials which is widely investigated for their attractive applications in electronic devices [6], super-capacitors [7,8], thermoelectric power generators [9], and gas and ion sensors [10,11,12]. Particularly, PPy and its derivatives play a leading role due to the relative low oxidation potential of the corresponding monomers, high stability and excellent electrical properties in organic and aqueous solvents [13]. Owing to these features, polypyrroles are considered as ideal polymeric materials for building various sensors.

In a global effort to combine the best of two worlds, PPy/CNT composites were prepared via chemical [14,15], electrochemical [16], sonochemical [17] and radiation-induced routes [18]. Indeed, strong interactions between the highly delocalized π-electrons of CNTs and π-electrons of the polymer skeleton favor the electron/hole transfer between CNTs and CPs [19,20]. It is worth to note that CNTs impart the polymer film electrical conductivity, which results in fast electrode kinetics [21]. Taking advantage of the salient and complementary features of CNTs and PPy, various electrochemical sensing systems based on PPy/CNT nanocomposites have been proposed for biomedical and environmental applications [22,23,24,25,26,27].

The main purpose of this work is to rationally modify flexible ITO electrodes with sensitive PPy films bearing embedded CNTs, for the selective recognition of heavy metallic ions. We have previously demonstrated that the excellent adhesion of PPy coating on flexible ITO required the use of coupling agents [28,29]. Otherwise, an immediate failure of the PPy coating occurred upon simple washing of the film with water. Sonication also induced a total delamination. Indeed, excellent adhesion was achieved on aminophenyl-modified ITO electrodes [28], as noted elsewhere for amino-modified materials [30]. For the preparation of composite films of PPy and CNTs, two options are proposed: Either polymerization of pyrrole on ITO electrode in the presence of CNT dispersion, or direct polymerization of pyrrole on CNT-coated ITO. This second option has been selected as first trials indicated robust CNT adhesion to arylated ITO surfaces (cf. Section 3).

Besides this interfacial aspect, in a previous paper, we reported on the doping of PPy with benzenesulfonic acid (BSA) and the remarkable electrochemical and electroanalytical properties of PPy [31]. Herein, we combine BSA with the strong ethylene glycol-bis(2-aminoethylether)-tetraacetic acid (EGTA) chelator [32]. The latter is similar to ethylenediaminetetraacetic acid (EDTA), but possesses an ethylene glycol unit. Since our ongoing research work targets the removal of metal ions, we reasoned that the EGTA chelator would add up two oxygen atoms with two electron pairs, and thus enhance the complexation of heavy metal ions. EDTA-grafted polypyrrole was previously employed for the selective electrochemical detection of heavy metals ions [33], while pyrrole was electropolymerized in the presence of single-walled CNTs and EDTA on stainless steel electrodes [34]. Herein, we conjugate the versatile surface chemistry of aryl diazonium salts on flexible ITO electrodes, CNTs and electrosynthesized PPy co-doped with BSA and EGTA. This allowed us to construct, in a unique manner, novel NH_2_-ITO/CNT/PPy electrodes for the capture of heavy metal ions. These flexible electrodes are easy to handle and to cut to the desired size with scissors.

The flexible sensing electrodes and reference materials were characterized using Raman, XPS, SEM and electrochemical impedance spectroscopy. Electroanalytical performances of the sensors were determined by differential pulsed voltammetry (DPV).

## 2. Experimental

### 2.1. Material and Reagents

CNTs of the type Nanocyl 7000, 2–9 nm diameter (carbon purity 99%), were purchased from Sigma Aldrich (St. Louis, MO, USA). Sodium nitrite (Sigma-Aldrich, ≥99.0%), anhydrous acetonitrile (99.8%, noted ACN), chloric acid, ferrocene (Sigma-Aldrich) and potassium chloride were used without further purification. Pyrrole (Aldrich, purity ≥98%), benzene sulfonic acid (BSA) and p-phenylenediamine were refrigerated prior to use. All aqueous solutions were prepared using Milli-Q ultrapure water (18 MΩ.cm). The flexible ITO-coated plastic sheets were purchased from Peccel (Yokohama, Japan).

### 2.2. In Situ Diazonium Modification of ITO Electrode

We have generated in situ para-aminobenzenediazonium cation from 1,4-phenylenediamine precursor in order to derivatize ITO sheets with surface bound aminophenyl groups (NH_2_-ITO). The ITO-coated polyethylene naphthalene (ITO-PEN) surface was cleaned by sonication in a solution containing water and ethanol for 2 min. After sonication, the electrode was washed with de-ionized water. The electrografting of diazonium salt generated in situ was performed using a three-electrode system, including a working electrode (ITO PEN, area ~5 × 5 mm), and a stainless-steel grid, used as counter electrode. All potentials were reported versus the saturated calomel electrode. The diazonium salt was produced by mixing 1 mM of diazonium with 0.5 M HCl in glassy beaker during 30 min before adding 10 mL of 1 mM aqueous solution of NaNO_2_.The resulting solution was stirred for 1 h. The electrografting was then carried out either by cyclic voltammetry (CV) at a scan rate of 50 mVs^−1^ or under potentiostatic conditions (−0.8 V/SCE for 45 s). We used the Biologic SP 150 potentiostat interfaced with the EC Lab software suite to conduct all electrochemical experiments. The blocking effect of the attached aminophenyl groups, as well as the electrochemical activity of the bare and modified ITO electrodes, were investigated by CV using a 1 mM Fe(CN)^3−/4−^ redox probe. The CVs were recorded between −0.4 and 0.6 V/SCE at a scan rate of 50 mV/s.

### 2.3. Purification and Deposition of CNTs on Diazonium-Modified ITO

The process of purification was adapted from the work of Bhakta et al. [35]. Briefly, 50 mg of crude CNTs were mixed with 20 mL of 6 M NaOH solution in a round-bottomed flask and then heated under constant agitation at 170 C for 1 h. The mixture was cooled, filtered and washed with copious amounts of water until neutral pH, and ultimately washed with acetone and dried in air.

In a second step, 25 mg of CNTs were dispersed in 37% HCl solution under reflux for 30 min [36]. The mixture was cooled, filtered and washed with water until neutral pH was obtained, and ultimately washed with acetone and dried in air. This oxidation procedure in concentrated acid is well known to provide carbon nanotubes with reactive carboxylic acid groups [37].

After activation, 1 mg of purified CNTs was dispersed by ultrasonication in 10 mL ACN for 1 h. The CNTs/NH_2_-ITO electrode surface was obtained by adding 50 µL of CNTs in ACN and evaporating the solvent after dropwise addition onto the NH_2_-ITO surface. We observed strong adherence of the sonicated CNTs on the NH_2_-ITO surface.

### 2.4. Preparation of PPy Films on ITO Surface

Prior to the electropolymerization, carbon nanotube (CNT)-decorated indium tin oxide (ITO) electrodes were sonicated in a solution containing water and ethanol for 2 min. Polypyrrole (PPy) was prepared by electropolymerization on bare ITO (Route 1), NH_2_-ITO (Route 2), CNT/NH_2_-ITO (Route 3) in the –1 to 1.2 V potential range, in an aqueous solution (~20 mL total volume) containing 0.01 M of bovine serum albumin (BSA), 0.01 M ethylene glycol-bis(2-aminoethylether)-tetraacetic acid (EGTA), 0.03 M NaOH and 0.1 M pyrrole.

### 2.5. Spectroscopic and Electrochemical Characterization of PPy-Modified Electrodes

Horiba Lab RAM HR Evolution was used to record Raman spectra at room temperature (RT). The beam wavelength was 638 nm.

X-ray photoelectron spectroscopy (XPS) measurements were carried out using K Alpha apparatus (Thermo Fisher Scientific, East Grinsted, UK) fitted with monochromated Al X-ray source (hv = 1486.6 eV; spot size = 400 µm). The pass energy was 80 and 200 eV for the narrow and the survey spectral regions, respectively. A flood gun was used for static charge compensation.

Electrochemical measurements were carried out using a Biologic SP105 potentiostat and EC-Lab as a monitoring software. The blocking effect of the electrochemical activity of the modified ITO surface was investigated by cyclic voltammetry (CV), using Fe(CN)^3−/4−^ redox couple and recorded between −0.4 and 1 V at a scan rate of 50 mV/s. The electrochemical impedance spectra (EIS) were recorded in the frequency range from 100 mHz–10 kHz. The equivalent electrical circuit was determined using EC-Lab software.

### 2.6. Morphological Characterization of PPy-Modified Electrodes

The films were imaged using a Field Emission Scanning Electron Microscope (Zeiss Merlin, Jena, Germany). Prior to analysis, all samples were coated with a 2 nm thin layer of platinum/palladium alloy using a Cressington 208HR sputter-coater coupled with a Cressington MTM-20 thickness controller. The CNT diameters were measured using an image analysis software (ImageJ, NIH). More than 100 measurements were performed for each sample in order to determine a CNT size distribution. Each measurement was marked with a white solid line.

## 3. Results and Discussion

Before reporting the results and discussing them, it is worth giving some useful information on the interfacial aspects of the actual sensors. The pending question was: is it necessary to apply an adhesive layer on ITO for the attachment of CNTs, or do they stick efficiently to bare ITO? The first trials demonstrated that CNTs had poor adhesion to bare ITO, a behavior similar to that of PPy. To address this limitation and overcome the adhesion problems, flexible ITO electrodes were modified with aminophenyl groups by the electroreduction of aminobenzenediazonium. We noted a better adhesion and resistance of CNTs to intensive washing with water and even to ultrasonication. This has motivated us to explore this route in view of building PPy/CNT composite coatings on flexible ITO in three steps: (i) preparation of aminophenyl-modified flexible ITO (NH_2_-ITO) [28], (ii) purification and ultrasonic dispersion of CNTs prior to deposition on NH_2_-ITO, (iii) electropolymerization of pyrrole in the presence of EGTA on CNT-coated NH_2_-ITO.

This strategy presents several advantages: CNTs were deposited on NH_2_-ITO by drop casting, which limits the loss of CNTs. Electropolymerization of pyrrole in a CNT suspension would have generated nanocomposites in suspension that would not have adhered to the surface by strong intermolecular interactions, therefore inducing detachment of the nanocomposite. Instead, coating CNTs on NH_2_-ITO is interesting, because the aminophenyl groups serve as coupling agents for CNTs through n–π electronic interactions. Indeed, sp^2^ carbon nanomaterials are known to have excellent adhesion to diazonium-modified surfaces [38].

With improved adhesion of CNTs to ITO through an aryl coupling agent monolayer, we anticipated obtaining strongly attached PPy to both the immobilized CNTs and the underlying aryl-modified ITO.

### 3.1. Preparation of PPy Films on Bare ITO, NH_2_-ITO and CNT/NH_2_-ITO Electrodes

**Scheme 1:**
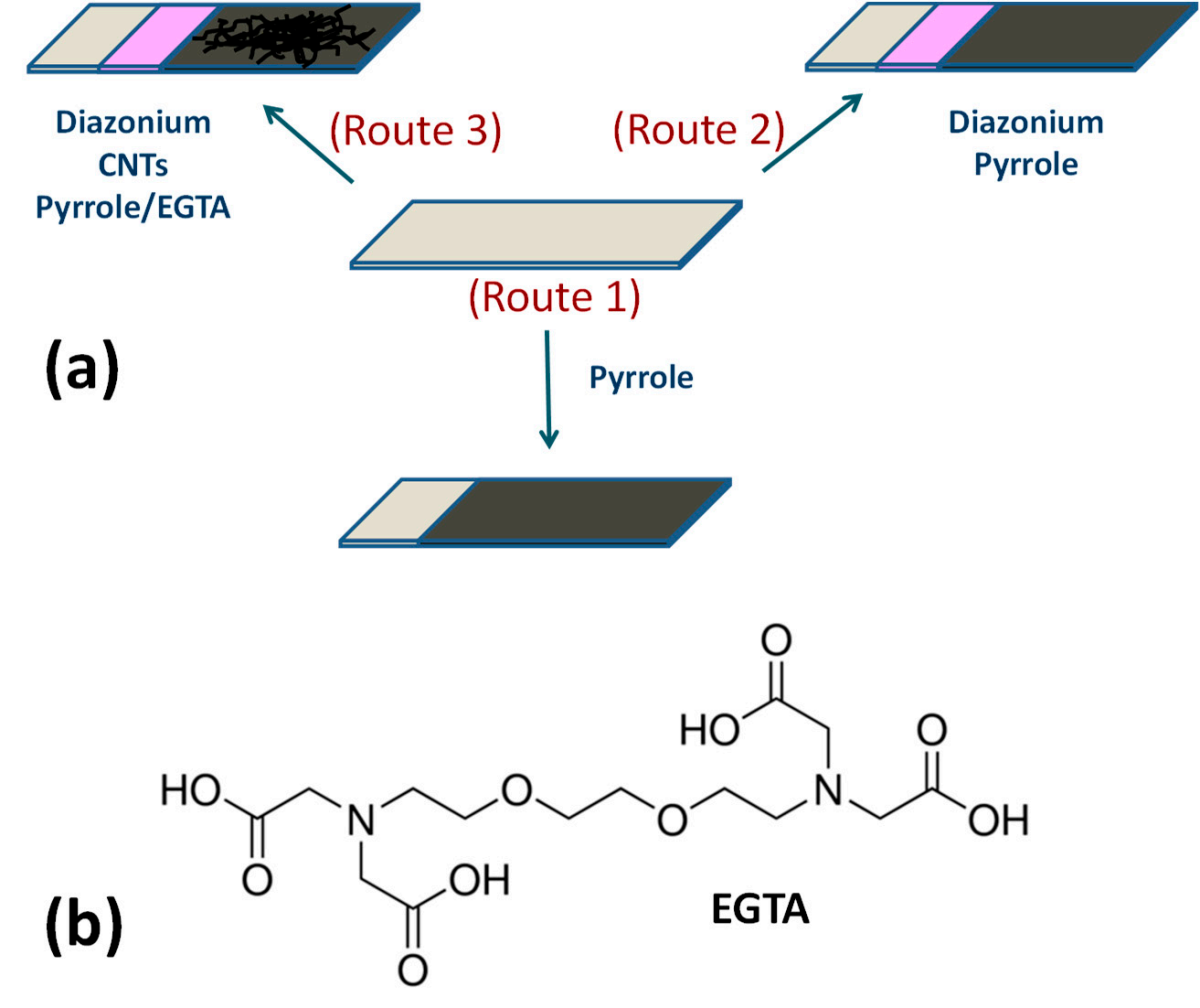
(**a**) Procedure for electrodeposition of polypyrrole on bare ITO (Route 1), NH_2_-ITO (Route 2), and CNT/NH_2_-ITO in the presence of ethylene glycol-bis(2-aminoethylether)-tetraacetic acid (EGTA) (Route 3), the structure of which is shown in (**b**).

Scheme 1 illustrates the strategies for making adhesively bonded PPy films with embedded CNTs on diazonium-modified flexible ITO sheets. The scheme also displays the preparation of reference materials, namely PPy-coated bare ITO and diazonium-modified ITO.

**Figure 1:**
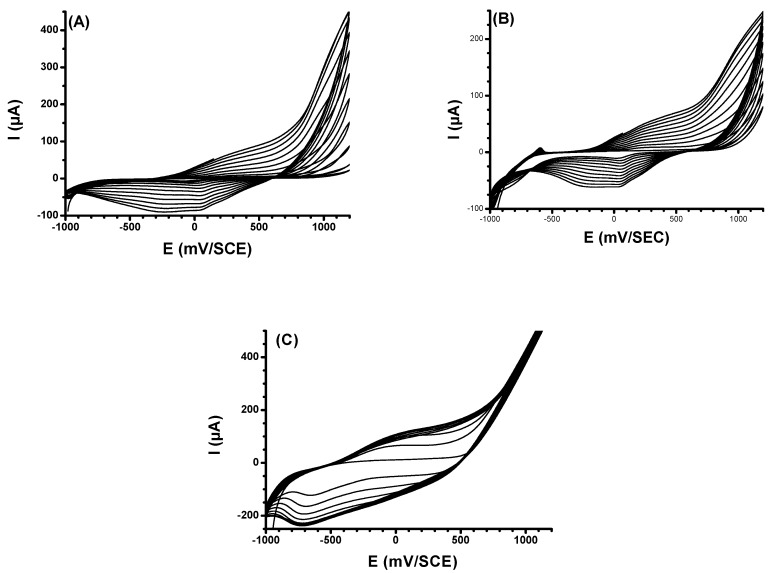
Electropolymerization of PPy on (**A**) bare ITO, (**B**) NH_2_-ITO and (**C**) CNTs/NH_2_-ITO.

Figure 1A–C represent the CVs obtained for the electrosynthesis in similar conditions of the PPy/ITO, PPy/NH_2_-ITO and PPy/CNT/NH_2_-ITO films, respectively. Cyclic voltammetry was performed for ten sequential cycles, at a scan rate of 0.1 V s^−1^, in the potential range (−1.0 to 1.2 V/SCE) in aqueous medium ~20 mL total volume containing 0.01 M of BSA, 0.01 M of EGTA and 0.03M of NaOH. The characteristic CV of pyrrole electropolymerization on bare ITO electrodes (Figure 1A) presents anodic and cathodic peaks at 0.34 and −0.25 V/SCE, respectively. The PPy film, prepared in these conditions on bare ITO, is not adherent, and could be easily removed, which contrasts with the remarkable adhesion of PPy films prepared on NH_2_-ITO (Figure 1B). The peak at ~+0.35 V/SCE corresponds to the oxidation of pyrrole on ITO-NH_2_, and increases with the number of cycles. The increase in the intensity of the oxidation and reduction peaks (at 0.4 and −0.12 V/SCE, respectively) with the number of cycles is consistent with the growth of an electroactive PPy coating [39]. Furthermore, the peak currents have higher intensities for the same system for PPy on a CNT/NH_2_-ITO surface (Figure 1C). The strongly adherent PPy films on CNT/NH_2_-ITO surfaces exhibit anodic and cathodic peaks at 0.25 and −0.85 V/SCE, which increases with the number of cycles. These differences in electroactivity versus the substrate nature clearly indicate that PPy coating deposited on CNT/NH_2_-ITO is the most conductive. These results are in line with those reported by Pilan et al. [40] in their study of polyaniline/carbon nanotube composite film electrosynthesis through diazonium salts electroreduction.

### 3.2. Structural Characterization of Modified Flexible ITO Sheets

#### 3.2.1. Electrochemical Properties

**Figure 2:**
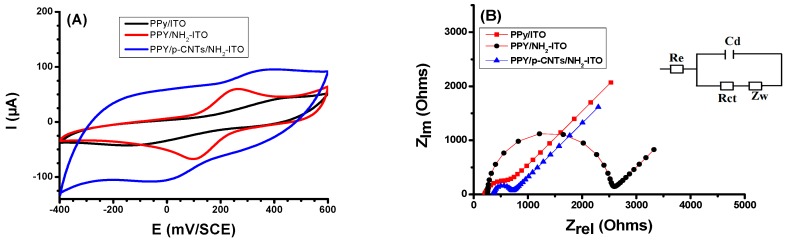
(**A**) Cyclic voltammograms, and (**B**) Nyquist plots of electrochemical impedance spectra obtained for PPy-ITO, PPy/NH_2_-ITO, PPy/CNTs/NH_2_-ITO. For CV: scan rate of 50 mV s^−1^; for EIS: within the frequency range of 100 mHz–10 kHz. Inset of Figure 2B displays the equivalent circuit. Cd: double layer capacitance; Rct: Charger transfer resistance; Zw: Warburg resistance; Re: resistance of the solution.

Fe(CN)^3−/4−^ was used as a redox probe to characterize PPy-ITO, PPy/NH_2_-ITO, PPy/CNT/NH_2_-ITO by CV and EIS (Figure 2). From CV (Figure 2A), one can extract the potential difference between the anodic and the cathodic peaks, which is high for PPy films deposited on bare ITO:

ΔE = | E_red_ − E_ox_ | = 430 mV

This difference can be linked to the absence of any adhesion between the PPy layer and the bare ITO surface [28]. In the case of PPy/NH_2_-ITO, the presence of −NH_2_ on ITO contributes to increase the electronic transfers leading to a lower ΔE (181 mV). For PPy/CNT/NH_2_-ITO, ΔE = 321 mV. The CVs are consistent with those obtained by EIS (Figure 2B). Indeed, EIS shows a low resistivity in the case of PPy/CNT/NH_2_-ITO, a significant blocking effect (increase of Rct) in the case of PPy-ITO, and a decrease of the resistivity character in the case PPy/NH_2_-ITO. The following Rct values were obtained: 2250, 690 and 445 Ohms for PPy/ITO, PPy/NH_2_-ITO and PPy/CNT/NH_2_-ITO, respectively.

The equivalent circuit diagram is shown in the inset of Figure 2B; it fits the three electrodes. It includes a charge transfer resistor (Rtc) describing the response of the film at high frequencies. The capacity of the electric double layer (Cd) takes into account the parasitic capacity of the ITO substrate. The circuit also contains an additional resistance (Re) in series with these elements which describes the resistance of the electrolytic medium. Finally, the constant phase element (Zw) in series with the film components describes the behavior of the ion blocking system near the electrodes.

From the above, the presence of a PPy layer on the CNT/NH_2_-ITO surface leads to an increase of electronic transfers due to the presence of the CNTs in the layer.

#### 3.2.2. Raman Study of Modified Flexible ITO Sheets

**Figure 3:**
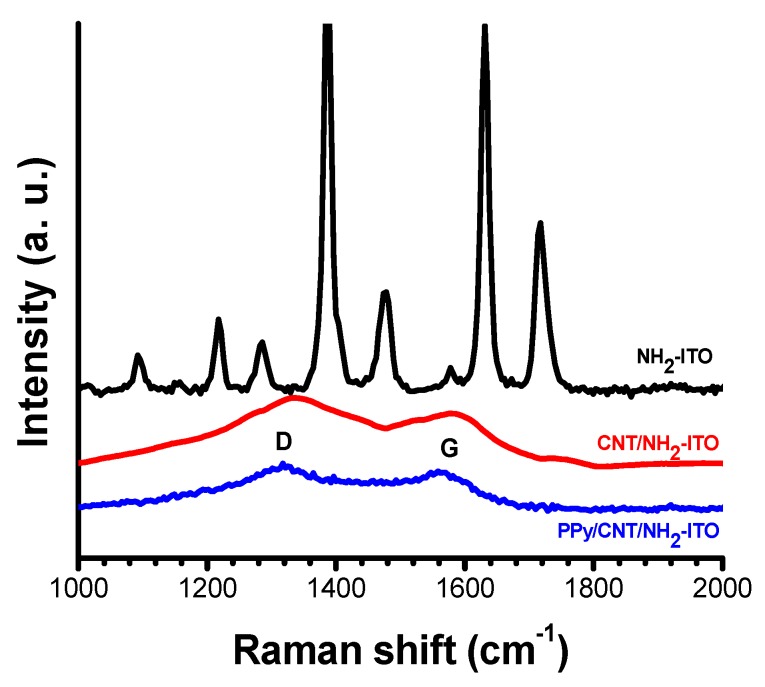
Raman spectra of NH_2_-ITO, CNT/NH_2_-ITO, PPy/CNT/NH_2_-ITO.

Figure 3 shows the Raman spectra of NH_2_-ITO, CNT/NH_2_-ITO and PPy/CNT/NH_2_-ITO. The NH_2_-ITO sample exhibits Raman bands due to the aryl groups, centered at 1387 and 1632 cm^−1^, assigned respectively to the C−H and C=C bonds in benzene ring. The band located at 1104 cm^−1^ is assigned to C–H in-plane vibrations and the band at 1282 cm^−1^ is due to the C−N stretching vibration [41].

The Raman spectra of CNTs deposited on the aryl diazonium layer show the most prominent features, i.e., the D band, associated with the defects in the hexagonal graphitic layers, and the G band, related to the vibrations of sp^2^-bonded carbon atoms in a two-dimensional hexagonal lattice. The D and G bands are centered at 1337 and 1587 cm^−1^, respectively, with the ID/IG peak intensity ratio of 1.21 for CNT/NH_2_-ITO. These results suggest that the surface treatment with the aryl diazonium salt does not affect the structure of the CNTs [17] and confirm that the aryl groups are interacting with CNTs via van der Waals interactions and H bonding.

The PPy/CNT/NH_2_-ITO surface exhibits a band at 1332 cm^−1^ attributed to the ring-stretching mode of PPy, whereas the peak at 1566 cm^−1^ is assigned to the backbone stretching mode of C=C bonds with the ID/IG peak intensity ratio 1.05 [42]. The Raman spectrum of PPy/CNT/NH_2_-ITO is similar to that obtained by Song et al. [42], and reveals that CNTs serve as the core in the formation of PPy/CNT composite.

#### 3.2.3. Morphology of Modified Flexible ITO Sheets

**Figure 4:**
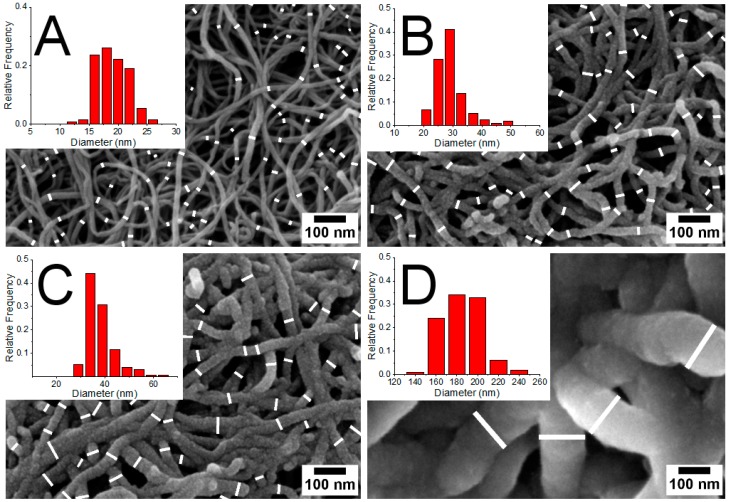
CNT size distribution. Histogram of the relative frequency versus the CNT diameters determined. (**A**) CNT/NH_2_-ITO, (**B**) PPy/CNT/NH_2_-ITO formed by CV for one cycle, (**C**) PPy/CNT/NH_2_-ITO formed by CV for five cycles and (**D**) PPy/CNT/NH_2_-ITO formed by CV for 10 cycles.

Figure 4A–D show SEM images of CNT/NH_2_-ITO and PPy/CNT/NH_2_-ITO, formed by CV for 1, 5 and 10 cycles. The SEM images of areas filled with CNTs (Figure 4A) further demonstrate that the tubes are homogeneous and can be distinguished individually with a nanometer scale width. In Figure 4B, the SEM images of PPy on CNT/NH_2_-ITO, that are formed after one CV, does not change the arrangement of the CNTs, but preferentially wraps around them, one by one. When the number of cycles increases to five (Figure 4C) and 10 (Figure 4D), the CNTs thicken gradually and homogeneously due to the wrapping by PPy. For 10 voltammetric cycles, PPy/CNT/NH_2_-ITO exhibits compact filaments with persisting three-dimensional porous microstructure [43]. Interestingly, there is no side deposition of PPy, indicating that the electrosynthesis of the polymer is confined to the sidewalls of the nanotubes, therefore suggesting strong interactions between PPy and the underlying CNTs.

CNTs diameter distributions for 0, 1, 5 and 10 CVs of PPy electrodeposition are shown in the insets of Figure 4A–D. The CNT average diameter is 18 ± 2.6 nm (Figure 4A), then increases to 27 ± 4.8, 35.6 ± 5.9 and 175 ± 20.1 after 1, 5 and 10 of electropolymerization of pyrrole, respectively (Figure 4B–D). The descriptive statistics are reported in Table 1. The SEM results confirm that the deposition of PPy is mostly confined to the CNT sidewalls.

**Figure 5:**
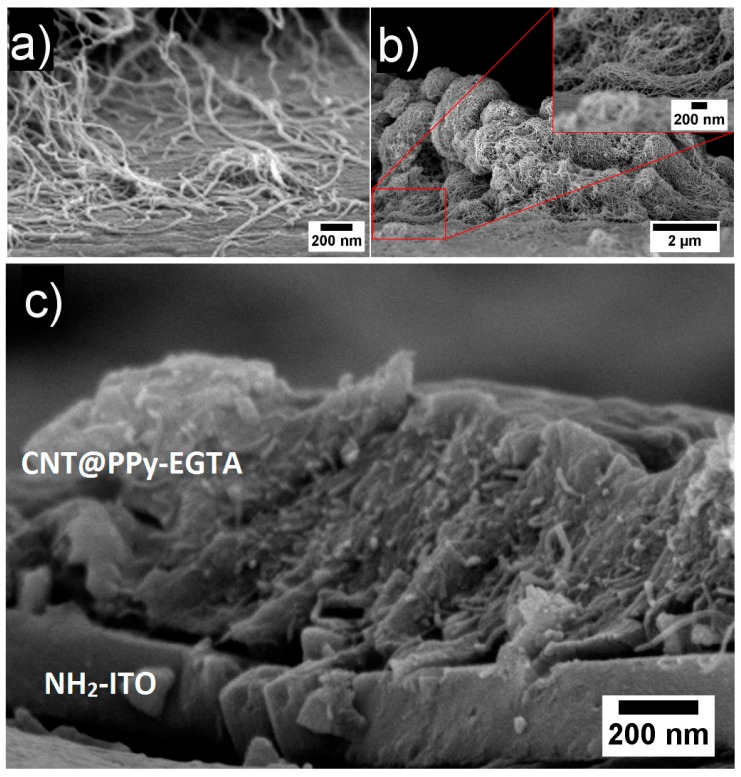
Scanning electron microscopy (SEM) images of (**a**) the interface between CNTs and NH_2_-ITO at high magnification (scale bar 200 nm) and (**b**) low magnification (scale bar 2 µm), (**c**) Interface between PPy-wrapped CNTs and NH_2_-ITO at high magnification (scale bar 200 nm). Inset of (**b**) shows front view of the cross-section at high magnification (scale bar 200 nm).

The interface between CNTs and NH_2_-ITO has been imaged by SEM (Figure 5). The attachment of CNTs on the NH_2_-ITO surface can be noted (Figure 5a,b), thus proving the interaction of the carbon nanotubes and diazonium-modified ITO [44,45]. The SEM morphology for the interface between PPy and CNT/NH_2_-ITO (Figure 5c) shows that PPy is wrapped around the CNTs [40], therefore confirming the structures displayed in Figure 4.

#### 3.2.4. XPS Surface Analysis

**Figure 6:**
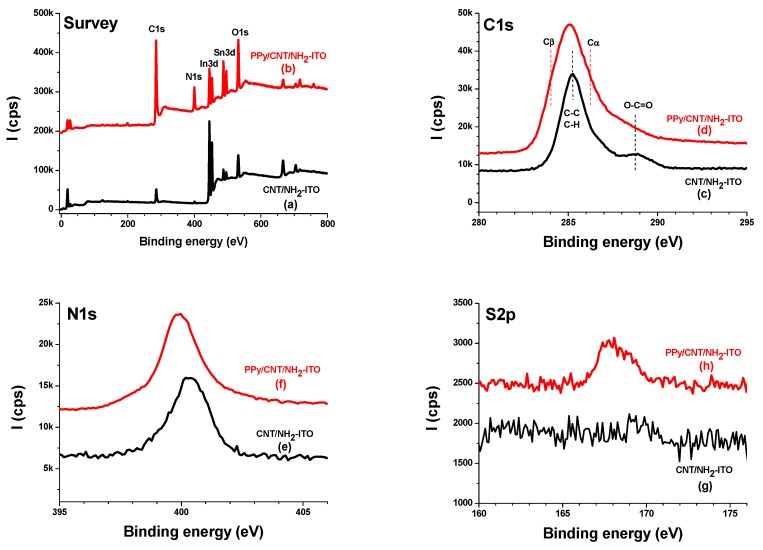
X-ray photoelectron spectroscopy (XPS) spectra of CNT/NH_2_-ITO (**a**,**c**,**e**,**g**) and PPy/CNT/NH_2_-ITO (**b**,**d**,**f**,**h**): Survey regions (**a**,**b**), C1s (**c**,**d**), N1s (**e**,**f**) and S2p (**g**,**h**).

We have previously reported the surface analysis of bare ITO and NH_2_-ITO electrodes [28,29,31]. Herein, we concentrate on CNT/NH_2_-ITO and PPy/CNT/NH_2_-ITO surfaces. Figure 6 displays survey, C1s, N1s and S2p spectra recorded for CNT/NH_2_-ITO and PPy/CNT/NH_2_-ITO. Figure 6a shows neat surface with C1s from CNTs on ITO with low C1s relative peak intensity, whilst C1s and N1s get much more intense after PPy coating (Figure 6b). The ITO is still visible (In3d at 445 eV) due to the porous nature of the sensing layer (see SEM Figure 4b,c). Nevertheless, the inelastic background exhibited in Figure 6b is in line with the ITO substrate screened by the PPy/CNT top layer. Concerning the narrow C1s regions (Figure 6c,d), it is clear that the shape changes significantly due to PPy; the broad spectrum has contributions from the carbon atoms in α and β positions to the nitrogen from the pyrrole ring [46]. Most probably, the shoulder noted at ~288.8 eV accounts for COO groups from EGTA. The N1s peak from CNT/NH_2_-ITO (Figure 6e) accounts for the aryl adhesive layer, whereas Figure 6f displays an N1s spectrum that has the shape of that previously reported for polypyrrole [47]. Finally, Figure 6g,h indicate that BSA is the only source of sulfur, and that the peak position accounts indeed for sulfur in a sulfonate chemical environment.

### 3.3. Electroanalytical Application of PPy/CNT/NH_2_-ITO Electrodes

**Figure 7:**
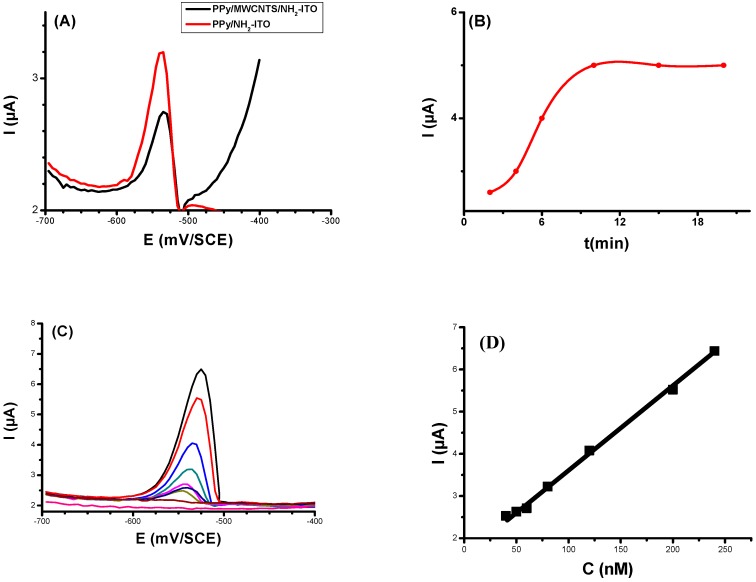
(**A**) Differential pulsed voltammetry (DPV) of Pb (II) at pH 5 containing Pb(II) 10^−7^ mol L^−1^ for PPy/CNT/NH_2_-ITO and PPy/NH_2_-ITO, (**B**) Time effect of pre-concentration metal ion on PPy/CNT/NH_2_-ITO, (**C**) Pb(II) stripping peak vs. Pb(II) concentration in the 10^−8^–2.5 × 10^−7^ mol L^−1^ range. (**D**) Calibration curve for the detection of Pb(II) using PPy/CNT/NH_2_-ITO electrode.

The electrochemical response was assessed in the same electrolytic solution containing 10^−7^ mol L^−1^ of Pb^2+^, using two different modified electrodes: PPy/CNT/NH_2_-ITO and PPy/NH_2_-ITO (Figure 7A). It is worth noting that the peak current obtained for PPy/CNT/NH_2_-ITO presents a higher value than that obtained for PPy/NH_2_-ITO due to the conductivity increase imparted by CNTs to the PPy matrix. As reported elsewhere, CNTs might enhance the diffusion rate of heavy metal ions during the sensing measurements [48].

The effect of pre-concentration time on the differential pulsed voltammetry (DPV) current is displayed in Figure 7B. The stripping peak current for both metals increased with the pre-concentration time up to 10 min. For periods longer than 10 min, the peak current was almost constant, which might be due to the electrode saturation by Pb^2+^. Therefore, the value of 10 min was chosen as the pre-concentration time.

PPy/CNT/NH_2_-ITO samples served to detect lead ions in an aqueous medium by differential pulse voltammetry (DPV) (Figure 7C). This figure displays DPV signals for Pb^2+^ concentrations in the 10^−8^ to 3 × 10^−7^ mol L^−1^ range (I (µA) = 1.5 C + 1.64, R = 0.996). The limit of detection (LOD) is deduced from the DPV peak intensity-vs-concentration correlation (Figure 7D); LOD = 2.9 × 10^−9^ mol L^−1^ and the limit of quantification LOQ = 8.7 × 10^−9^ mol L^−1^ at S/N = 3. It is worth to note that the actual LOD is lower than that reported for EDTA-functionalized polypyrrole (0.1 ppm) [33] and for EDTA-PPy/SWNTs nanocomposite (70 × 10^−9^ mol L^−1^) [34].

**Figure 8:**
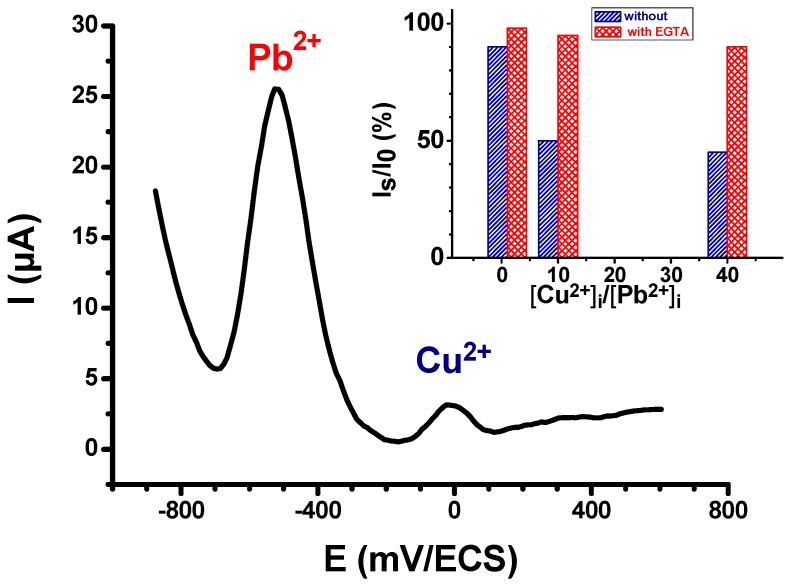
DPV simultaneous determination of concentration 2.510^−6^ M of a Pb^2+^ and Cu^2+^ mixture with initial Cu^2+^/Pb^2+^ molar ratio = 1. Inset shows that for [Cu^2+^]_i_/[Pb^2+^]_i_ = 1, 10, 40, I_s_/I_0_ = 90, 50 and 45, respectively, without EGTA; whilst I_s_/I_0_ = 98, 95 and 90, respectively, in the presence of EGTA.

At this stage, the question concerning the use of EGTA remains open: how useful is this chelator? Previously, we have shown that without any CNTs and EGTA, the LOD is ~1 × 10^−9^ mol L^−1^, which is slightly better. However, with CNTs, a better conductivity of the sensing top layer is obtained. Concerning EGTA, we have investigated the selectivity of the film towards Pb^2+^ and Cu^2+^. According to our previously published procedure [31], we have determined, for a total initial concentration of metal ions of 2.5 × 10^−6^ mol L^−1^, the relative intensity of the Pb^2+^ stripping peak, in the presence and in the absence of interfering Cu^2+^ ions, at various initial Cu^2+^/Pb^2+^ molar ratios. The electrochemical response is displayed in Figure 8. As it can be seen, there are two well-resolved stripping peaks assigned to Pb^2+^ and to Cu^2+^, respectively. The inset of Figure 8 plots the Pb peak intensity ratio I_s_/I_0_ (in %), where I_s_ and I_0_ stand for the stripping peak of Pb^2+^ in the presence and absence of Cu^2+^ interfering cations, respectively. Obviously, with EGTA, despite the presence of Cu^2+^, the stripping response of Pb^2+^ does not decrease significantly, hence the interest of co-doping PPy with the EGTA chelator.

### 3.4. Density Functional Theory Calculations

Quantum chemical calculations were performed using density functional theory (DFT) at the B3LYP/6-31+G* level. The 6-311+G(d) basis set was used for H, C, N, O atoms and the LANL2DZ basis set for Cu and Pb atoms as implemented in Gaussian16 [49,50].

**Scheme 2:**
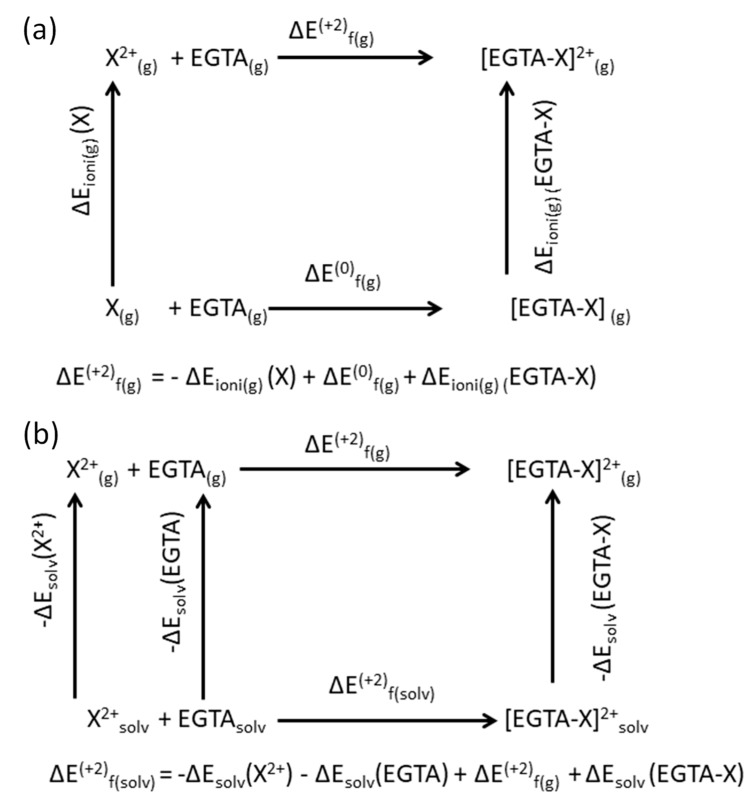
Thermodynamic cycles used to calculate the complexation energy in gas (**a**) and in solvent (**b**).

The solvent effect was added by using the United Atom molecular cavity model UAKS (united atom Kohn–Sham model) [51]. The formation energy of the complexes in gas phase and in solvent was computed from Hess thermodynamic cycle (Scheme 2). The gas phase ionization energy was extracted from the paper published by Barone et al. [52]. 

**Figure 9:**
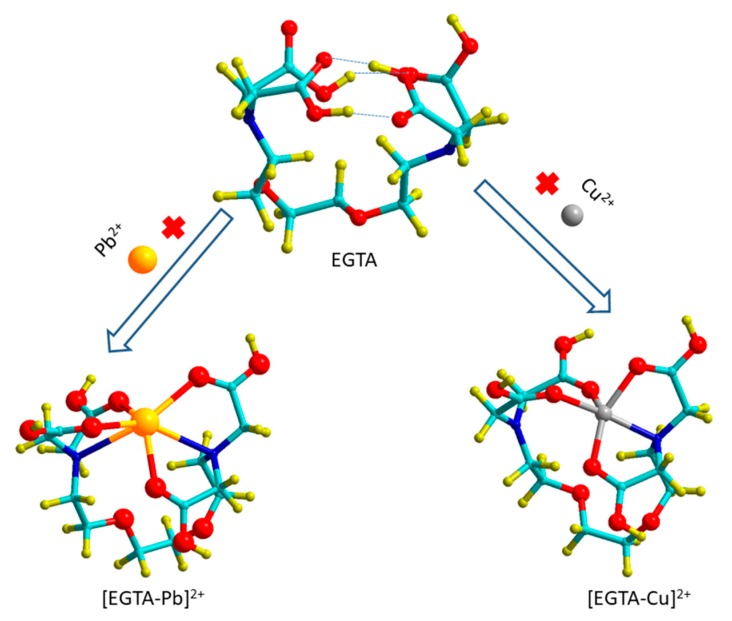
Structures of stable conformers of EGTA and, [EGTA-Pb]^2+^ and [EGTA-Cu]^2+^ complexes.

An exhaustive conformational search was explored for the EGTA molecule in order to determine the most stable structure. The most stable configuration is stabilized by hydrogen bonds between carboxylic acid groups, as it can be seen in Figure 9. This conformer is used to complex with Cu^2+^ and Pb^2+^ ions. The formed complexes are fully optimized, and the formation energies are computed in both gas and liquid phases. In gas phase, the calculated complexation energy was found to be –802.4 kJ/mol and –741.9 kJ/mol for Pb^2+^ and Cu^2+^, respectively. When adding the solvent effect, the values decrease to −374.6 and −116.4 kJ/mol for Pb^2+^ and Cu^2+^, respectively. The difference is linked to the more favorable complexation in the gas phase of Pb^2+^ and the important solvation of Cu^2+^ (Table 2). Based on these thermodynamics results, one can conclude that the formation of the Pb^2+^ complex is more favorable compared to Cu^2+^. The optimized geometries of these complexes are displayed in Figure 9 and show different types of coordination for the two metal ions. The Pb^2+^ ion is hexa-coordinated with four O–Pb bonds, the length of which varying between 2.452 and 2.662 Å, i.e., close to the sum of the Pb and O atomic radii (2.44 Å). It is bonded to nitrogen atoms at distances of 2.854 and 2.903 Å, larger than the sum of the atomic radii of isolated elements. The Cu^2+^ ion is penta-coordinated with four oxygen and one nitrogen atoms. The bond length of Cu−O varies between 2.04 and 2.23 Å, close to the sum of the atomic radii of Cu and O (2.01 Å). The Cu−N bond length is 2.13 Å, larger than the sum of the atomic radii of Cu and N (2.03 Å).

## 4. Conclusions

PPy/CNT/NH_2_-ITO composites were prepared by electropolymerization on diazonium-modified flexible ITO electrodes coated with CNTs, in the presence of the EGTA chelator. Polypyrrole was also prepared by electropolymerization on NH_2_-ITO and on bare ITO, without any CNTs. CNTs were easily removed from bare ITO, but resisted solvent washing on NH_2_-ITO-flexible electrodes. Electrodeposition of PPy was found to be confined to the CNTs sidewalls, as evidenced by SEM and the growth of the CNT diameter with the number of voltammetric cycles used to generate polypyrrole. The electropolymerization of PPy on the CNT surface exhibits more expanded molecular conformation and more ordered chain packing, due to the strong π–π and n–π conjugation interactions between PPy and CNTs and the efficient template effect of CNTs during the electropolymerization. The increase of the electrochemical conductivity has been observed in the PPy deposited on CNT/NH_2_-ITO.

The PPy/CNT/NH_2_-ITO composite electrodes were used for the electroanalysis of Pb^2+^ in aqueous media. The LOD was found to be 2.9 × 10^−9^ mol L^−1^ at S/N = 3. Interestingly, EGTA was found to drastically improve the selectivity of the sensor towards Pb^2+^, as the response of the EGTA-containing sensor did not change, even in the presence of a relatively large concentration of competitive Cu^2+^ metal ions. Such improved selectivity imparted by EGTA is due to the higher EGTA-Pb^2+^ interaction energy (−374.6 kJ/mol) compared to that of EGTA-Cu^2+^ (−116.4 kJ/mol), as judged from DFT calculations that consider solvation effects.

This work demonstrates conclusively the importance of diazonium salts to improve CNTs adhesion to ITO electrode surfaces, serving as template for the in situ polypyrrole electrodeposition. The embedded CNTs impart remarkable properties to the polypyrrole sensing layers, the selectivity of which is drastically improved using the EGTA chelator.

## Figures and Tables

**Table 1:** Average CNT diameters and descriptive statistics summary.

Sample	Mean	Standard Deviation	Median	Minimum	Maximum
	(nm)	(nm)	(nm)	(nm)	(nm)
CNT/NH_2_-ITO	18.0	2.6	17.9	11.0	24.8
PPy/CNT/NH_2_-ITO	27.0	4.8	26.1	19.8	46.5
formed by CV for 1 cycle					
PPy/CNT/NH_2_-ITO	35.6	5.9	34.6	25.1	63.0
formed by CV for 5 cycles					
PPy/CNT/NH_2_-ITO	175.0	20.1	176.5	126.3	233.0
formed by CV for 10 cycles					

**Table 2:** Formation energies in gas phase (∆E^(+2)^_f(g)_), in solvent (∆E^(+2)^_f(solv)_), solvation energies and X-O and X-N minimum and maximum distances for the most stable complexes.

System	∆E^(+2)^_f(g)_	∆E_solv_	∆E^(+2)^_f(solv)_	dX-O (Å)	dX-N (Å)
	(kJ/mol)	(kJ/mol)	(kJ/mol)		
EGTA	-	−127.8	−	-	-
Cu^2+^	-	−1288.8	−	-	-
Pb^2+^	-	−1038.8	−	-	-
[EGTA-Cu]^2+^	−741.9	−790.9	−116.4	2.047 (2.238)	2.135 (3.574)
[EGTA-Pb]^2+^	−802.4	−738.8	−374.6	2.452 (2.662)	2.854(2.903)
